# College and Hospital Notes

**Published:** 1902-10

**Authors:** 


### COLLEGE AND HOSPITAL NOTES.

THE Cincinnati College of Medicine and Surgery has closed its doors, for lack of sufficient support. This would appear to be the direct result of the requirement on the part of the state of higher standards for entrance upon the study of medicine. Following the lead of New York many states, Ohio included, have established a rigid system of entrance examinations, and others are year by year adopting similar measures. The effect of this will be to strengthen the strong colleges and weaken the weak ones, until finally, let it be hoped, there will be uniformity of standards throughout the United States.

---

The Medical department of the University of Buffalo began its fifty-seventh annual session September 29, 1902, to continue thirty-two weeks. The opening lecture was delivered by Dr. Lucien Howe, professor of ophthalmology. The number in attendance was large, and the university is in a prosperous condition.

---

The consulting staff of the Emergency Hospital for the ensuing year has been appointed as follows: president, Dr. John H. Pryor; vice-president, Dr. Wm. C. Krauss; secretary, Dr. Vertner Kenerson.

---

The Buffalo Clinical Laboratory has been organised to meet the demands for clinical work at rates within the means of all classes, equipping one centrally located laboratory where a small staff of workers can carry on clinical examinations for a large number of physicians. By this means busy doctors have the advantages of reliable clinical analysis of blood, urine, and the like, and prompt reports. For the convenience of patrons the laboratory will maintain a messenger service and will send for urine and other clinical material to any place within the city limits without extra charge.

An institution of this sort demands a considerable initial expenditure in order to get ready for work. It is proposed to raise a capital of $5,000 for the equipment of the laboratory and purchase of supplies. The sum is divided into 500 shares of $10 each. When the requisite number of shares have been paid into the treasury, the laboratory will be incorporated under the laws of New York, thus acquiring the legal standing and permanency that are desirable in order to do business on a good business basis. It will then be ready to begin work.

Physicians are appealed to personally to assist in this most desirable enterprise by subscribing for as many shares as each may choose, and it is further urged that subscriptions be made as promptly as possible, in order that there may be no delay in opening the laboratory and beginning the work. Subscriptions or enquiries may be addressed to any of the officers who are as follows: president, J. H. Potter; secretary, A. L. Benedict; treasurer, H. C. Rooth; director, Franklin W. Barrows; assistant director, William Irving Thornton.

